# Supplementary material for: Prevalence and Molecular Characteristics of FAdV-4 from Indigenous Chicken Breeds in Yunnan Province, Southwestern China
Source: Microorganisms. 2023 Oct 26;11(11):2631. doi: 10.3390/microorganisms11112631 (PMC10673041; doi:10.3390/microorganisms11112631)
Supplement: Supplementary file 1 [file microorganisms-11-02631-s001.zip › Table S1.Primers were used in study.pdf]

Table S1. Primers used to amplify the capsid protein sequence of FAdV strains

PCR product	Sequences of primers	Products size	Reference
FAdV-JD	FAdV-JD-F: CAAGTTCAGACAGACGGT	889 bp	
	FAdV-JD-R: AGTGATGACGGGACATCAT		
Hexon	Hexon-F1: CGTCTAGGTTCGCACCGCCATGGC	1501 bp	
	Hexon-R1: CATCTGGTCGATGGACCAACGCGCACC		
	Hexon-F2: CATCGACCAGATGGACAACGTCAACCCCTTCAAC	1345 bp	[1]
	Hexon-R2: TTACACGGCGTTGCCTGTGGCG		
Fiber	C-Fib1-F: AGATCTATGTCGGCCCTAATC	1287 bp	
	C-Fib1-R: AAGCTTTTAGGGGCTCGGAGC		[2]
	C-Fib2-F: AGATCGATGCTCCGAGCCCCTAA	1426 bp	
	C-Fib2-R: CGGCCGTTACGGGAGGGAGCCC		
Penton	C-Penton-F: GGATCCATGTGGGGGTTGCAGCCGCCGACGT	1577 bp	
	C-Penton-R: CTCGAGCTACTGCAAGGTCGCGGAACTCAGA		

1. Chen, L.; Yin, L.; Zhou, Q.; Peng, P.; Du, Y.; Liu, L.; Zhang, Y.; Xue, C.; Cao, Y. Epidemiological investigation of fowl adenovirus infections in poultry in China during 2015-2018. *BMC veterinary research* **2019**, *15*, 271, doi:10.1186/s12917-019-1969-7.
2. Marek, A.; Nolte, V.; Schachner, A.; Berger, E.; Schlötterer, C.; Hess, M. Two fiber genes of nearly equal lengths are a common and distinctive feature of Fowl adenovirus C members. *Veterinary microbiology* **2012**, *156*, 411-417, doi:10.1016/j.vetmic.2011.11.003.
