# Supplementary material for: Prevalence and Molecular Characteristics of FAdV-4 from Indigenous Chicken Breeds in Yunnan Province, Southwestern China
Source: Microorganisms. 2023 Oct 26;11(11):2631. doi: 10.3390/microorganisms11112631 (PMC10673041; doi:10.3390/microorganisms11112631)
Supplement: Supplementary file 1 [file microorganisms-11-02631-s001.zip › Table S2.Details of fowl adenovirus isolates for sequence alignment and phylogenetic analysis;.pdf]

Table S2. Details of fowl adenovirus isolates for sequence alignment and phylogenetic analysis

Species	Accession no	Name of strains	origin	date of collection
	MF168407.1	JM1/1	Japan	Nov-00
Fowl adenovirus A	NC_001720.1	VR-432	Phelps	Jan-96
	NC_021221.1	FAdV5-340	Ireland	Early 1970s
Fowl adenovirus B	OK283055.1	19/7209	Hungary	2019
	KT717889.1	C-2B	USA	Sep-15
	KU877435.1	SDSX-15	China	20-MAR-2016
	KU877420.1	HBLF-15	China	20-MAR-2016
	KU877419.1	SDFC2-15	China	20-MAR-2016
	KX364100.1	SDTW2-16	China	03-APR-2017
	MK875249.1	CH/GDHZ-S7/2017	China	24-NOV-2019
	MK875247.1	CH/GDBL-S2/2017	China	24-NOV-2019
	KU877436.1	SDBZ-15	China	20-MAR-2016
	KU877427.1	SDXT4-15	China	20-MAR-2016
	KU877426.1	SDDM-15	China	20-MAR-2016
	KU877416.1	SDSK-15	China	20-MAR-2016
	KU877432.1	SDXT2-15	China	20-MAR-2016
	KU877422.1	SDXT8-15	China	20-MAR-2016
	KU877417.1	SDZB-15	China	20-MAR-2016
	KU877434.1	SDJN1-15	China	20-MAR-2016
	KU877429.1	SDXT3-15	China	20-MAR-2016
	KU877421.1	SDJN2-15	China	20-MAR-2016
	KU877415.1	SDFC4-15	China	20-MAR-2016
Fowl adenovirus C.	OP917885.1	SDRZ-21072101	China	16-JAN-2023
	MZ435838.1	CH/JSXZ/1905	China	19-OCT-2022
	KU647684.1	CG-G	China	08-AUG-2016
	MG148335.1	CH/AHMC/2015	China	09-SEP-2018
	ON929900.1	SDLY	China	12-OCT-2022
	ON652872.1	Pasouk	Astrulian	08-SEP-2022
	AJ459805.1	Indian	Indian	15-APR-2005
	EU931692.1	PJ-06	Indian	02-SEP-2008
	KT717889.1	C-2B	USA	04-AUG-2021
	KU175344.1	FAdV/PA/Layer/27614/13	USA	02-MAY-2016
	HQ709227.1	Kr-Gunwi	South Korea	21-FEB-2017
	GU188428.1	ON1	Canada	26-MAY-2011
	KP295475.1	MX-SHP95	Mexico	29-JUN-2015
	KU981155.1	FAdV-HBSJZ-14094-O	China	31-MAR-2017
	KU981155.1	FAdV-HBSJZ-14094-O	China	31-MAR-2017
		YNBJ-5/202305	China	2023
		YNBL-8/202303	China	2023
		YNBJ-5/202305	China	2023

		YNBL-47/202303	China	2023
		YNFD-202211	China	2022
		YNJC-202212	China	2022
		YNLP-202208	China	2022
		YNNJ-202207	China	2022
		YNXJ-202210	China	2022
		YNYL-202205	China	2022
		YN-NC1/202203	China	2022
	KU746335.1	MX95-S11	Mexico	January-1995
Fowl adenovirus D	AC_000013.1	Fowl adenovirus D	unknown	2018
	JN112373.1	764	Canada	Dec-09
Fowl adenovirus E	KX077988.1	HLJ/151129	China	2015
	KU517714.1	UPM04217	Malaysia	Dec-09
