# Supplementary material for: Prevalence and Molecular Characteristics of FAdV-4 from Indigenous Chicken Breeds in Yunnan Province, Southwestern China
Source: Microorganisms. 2023 Oct 26;11(11):2631. doi: 10.3390/microorganisms11112631 (PMC10673041; doi:10.3390/microorganisms11112631)
Supplement: Supplementary file 1 [file microorganisms-11-02631-s001.zip › Table S3.Information on strains with the highest homology to FAdV-4 strains isolated in our study..pdf]

Table S3 Information on strains with the highest homology to FAdV in Southwest China.

Isolate	Similarity with other Chinese isolates at the Nucleotide level				Similarity with other Chinese isolates at the Nucleotide level			
	Strain	Accession no	Similarity（%）	Location	Strain	Accession no	Similarity（%）	Location
YNBL-8/202303	GDMZ	MG856954.1	99.72	Guangdong	ZJ2015	MF521611.1	99.72	Zhejiang
	ZZ	MN337322.1	99.72	Henan	GDYF	MK387062.1	99.72	Guangdong
	SCDY	MK629523.1	99.72	Sichuan	SD16101/FAdV-4	MH006602.1	99.72	Shandong
	GX-1	MH454598.1	99.72	Guangxi	CH/AHMC/2015	MG148335.1	99.72	AnHui
YNBJ-5/202305	GDMZ	MG856954.1	99.86	Guangdong	HLJDAd15	KX538980.1	99.86	Heilongjiang
	ZZ	MN337322.1	99.86	Henan	GX-1	MH454598.1	99.86	Guangxi
	SCDY	MK629523.1	99.86	Sichuan	SD16101/FAdV-4	MH006602.1	99.86	ShanDong
	GDYF	MK387062.1	99.86	Guangdong				
YNBL-7/202303	GDMZ	MG856954.1	99.72	Guangdong	GX2020-019	OP378126.1	99.72	Guangxi
	SCDY	MK629523.1	99.72	Sichuan	SD1601/FAdV-4	MH006602.1	99.72	ShanDong
	AH712	KY436522.1	99.72	Anhui	HeBei/0715/2021	OP223055.1	99.72	HeBei
	HLJDAd15	KX538980.1	99.72	Heilongjiang				
YNFD/202211	GDMZ	MG856954.1	99.90	Guangdong	CH/JSXZ/2015	KU569296.1	99.90	Jiangsu
	SCDY	MK629523.1	99.90	Sichuan	HLJ/160826	KY569422.1	99.90	HeiLongjiang
	AH712	KY436522.1	99.90	Anhui				
	SD1511	MF496037.1	99.90	Shandong				
YNJC/202212	GDMZ	MG856954.1	100.00	Guangdong	CH/GDYF/201706	MK387062.1	100.00	GuangDong
	SCDY	MK629523.1	100.00	Sichuan	AH712	KY436522.1	100.00	AnHui
	CH/JSXZ/2015	KU569296.1	100.00	Jiangsu				
	GX2017-01	MN577977.1	100.00	Guangxi				
YNLP/202208	GDMZ	MG856954.1	99.82	Guangdong	PDS	KU647685.1	99.82	Henan
	SCDY	MK629523.1	99.82	Sichuan	CH/AHMC/2015	MG148335.1	99.82	AnHui
	HLJ/151118	KX061750.1	99.82	Heilongjiang	CH/GDYF/201706	MK387062.1	99.82	GuangDong
	AH712	KY436522.1	99.82	Anhui				
YN-NC1/202203	GDMZ	MG856954.1	99.97	Guangdong	HeBei/0914/2021	OP223053.1	99.97	Hebei
	SCDY	MK629523.1	99.97	Sichuan	CH/GDYF/201706	MK387062.1	99.97	GuangDong
	AH712	KY436522.1	99.97	Anhui	CH/AHMC/2015	MG148335.1	99.97	AnHui
	GX2020-019	OP378126.1	99.97	Guangxi				
YNNJ/202207	GDMZ	MG856954.1	99.90	Guangdong	GX2020-019	OP378126.1	99.90	Guangxi
	SCDY	MK629523.1	99.90	Sichuan	CH/GDYF/201706	MK387062.1	99.90	GuangDong
	AH712	KY436522.1	99.90	Anhui	CH/AHMC/2015	MG148335.1	99.90	AnHui
	HLJDAd15	KX538980.1	99.90	Heilongjiang				
YNXJ/202210	GDMZ	MG856954.1	99.89	Guangdong	HeBei/0914/2021	OP223053.1	99.89	Hebei
	SCDY	MK629523.1	99.89	Sichuan	CH/GDYF/201706	MK387062.1	99.89	GuangDong
	AH712	KY436522.1	99.89	Anhui	CH/AHMC/2015	MG148335.1	99.89	AnHui
	HLJFAd15	KU991797.1	99.89	Heilongjiang	PDS	KU647685.1	99.89	HeNan
YNYL/202205	GDMZ	MG856954.1	99.93	Guangdong	SDSX1	KU845700.1	99.93	Shandong

SCDY	MK629523.1	99.93	Sichuan	CH/GDYF/201706	MK387062.1	99.93	GuangDong
AH712	KY436522.1	99.93	Anhui	CH/AHMC/2015	MG148335.1	99.93	AnHui
HLJ/160826	KY569422.1	99.93	Heilongjiang				
